# *Bacteroides fragilis* alleviates necrotizing enterocolitis through restoring bile acid metabolism balance using bile salt hydrolase and inhibiting FXR-NLRP3 signaling pathway

**DOI:** 10.1080/19490976.2024.2379566

**Published:** 2024-07-16

**Authors:** Zhenhui Chen, Huijuan Chen, Wanwen Huang, Xiaotong Guo, Lu Yu, Jiamin Shan, Xiaoshi Deng, Jiaxin Liu, Wendan Li, Wei Shen, Hongying Fan

**Affiliations:** a Department of Microbiology, Guangdong Provincial Key Laboratory of Tropical Disease Research, School of Public Health, Southern Medical University, Guangzhou, China; b Experimental Teaching Center of Preventive Medicine, Guangdong Provincial Key Laboratory of Tropical Disease Research, School of Public Health, Southern Medical University, Guangzhou, China; c Department of Radiation Oncology, Nanfang Hospital, Southern Medical University, Guangzhou, China; d Department of Neonatology, Nanfang Hospital, Southern Medical University, Guangzhou, China

**Keywords:** Necrotizing enterocolitis, bile acid metabolism, FXR, NLRP3, *B. fragilis*, BSH, Organoids

## Abstract

Necrotizing enterocolitis (NEC) is a leading cause of morbidity and mortality in premature infants with no specific treatments available. We aimed to identify the molecular mechanisms underlying NEC and investigate the therapeutic effects of *Bacteroides fragilis* on NEC. Clinical samples of infant feces, bile acid-targeted metabolomics, pathological staining, bioinformatics analysis, NEC rat model, and co-immunoprecipitation were used to explore the pathogenesis of NEC. Taxonomic characterization of the bile salt hydrolase (*bsh*) gene, enzyme activity assays, 16S rRNA sequencing, and organoids were used to explore the therapeutic effects of *B. fragilis* on NEC-related intestinal damage. Clinical samples, NEC rat models, and in vitro experiments revealed that total bile acid increased in the blood but decreased in feces. Moreover, the levels of FXR and other bile acid metabolism-related genes were abnormal, resulting in disordered bile acid metabolism in NEC. Taurochenodeoxycholic acid accelerated NEC pathogenesis and taurodeoxycholate alleviated NEC. *B. fragilis* displayed *bsh* genes and enzyme activity and alleviated intestinal damage by restoring gut microbiota dysbiosis and bile acid metabolism abnormalities by inhibiting the FXR-NLRP3 signaling pathway. Our results provide valuable insights into the therapeutic role of *B. fragilis* in NEC. Administering *B. fragilis* may substantially alleviate intestinal damage in NEC.

## Introduction

Necrotizing enterocolitis (NEC) is an intestinal inflammatory disease that can develop into intestinal necrosis and sepsis, and a significant cause of morbidity and mortality in neonates in intensive care units.^1^ The incidence rate of premature NEC with a weight of less than 1500 g is up to 7%, and the fatality rate is 20%–30%.^2^ In addition, 25% of surviving children exhibit severe sequelae such as intestinal stenosis and growth retardation.^3^ Several studies have shown that NEC is closely associated with gut microbiota dysbiosis.^4,5^ Disorders of the intestinal microbiota activate a series of cytokines, destroy the intestinal mucosal barrier, and cause an excessive inflammatory response in the intestinal wall tissue, thereby resulting in local or diffuse necrosis of the intestinal epithelial cells.^6^ However, the pathogenic role of the gut microbiota in NEC has not been fully clarified.

Because bile acid metabolism is associated with the gut microbiota and intestinal immunity, it has attracted the interest of researchers studying NEC. Bile acid metabolism bridges the gut microbiota and host.^7^ The process of bile acid metabolism in the intestinal tract is complex and requires the participation of two types of bacteria: those with bile salt hydrolase (BSH) and 7α-dehydroxylation enzymes. *Bacteroides*, *Lactobacillus*, and *Bifidobacterium* are reported to contain BSH. These microorganisms convert primary conjugated bile acids into deconjugated bile acids, which subsequently perform 7α-dehydroxylation to form secondary bile acids.^8,9^ Disorders of the gut microbiota result in abnormal bile acid metabolism, thus damaging the normal barrier function and homeostasis of the host and promoting the occurrence and development of inflammation and gastrointestinal diseases.^10–12^ Bile acid metabolism is also involved in the pathogenic process of NEC and a significant elevation of total bile acid (BA) levels is observed in patients with NEC.^13^ Reduced colonization of intestinal flora and formula feeding promote toxic bile acid accumulation, exacerbating intestinal injury in severe NEC.^14^ Moreover, total bile acid levels in the ileum and NEC incidence/severity are positively correlated, with bile acid reduction upon NEC relief.^15^ However, whether NEC can be cured by regulating bile acid metabolism through probiotics or gut microbes remains unclear.

The Farnesoid X receptor (FXR) is a bile acid-regulated transcription factor and a protective sensor of hepatocytes and gastrointestinal tissues that is highly expressed in the intestine and liver.^16^ FXRs are crucial components in regulating metabolism and the immune system.^17^ Recent studies have revealed surprising functions of FXR, including intestinal barrier protection, innate immune development, and carcinogenesis, indicating that FXR may be a promising therapeutic target for intestinal diseases.^18–20^ FXR agonists have been used to treat mice with lipopolysaccharide (LPS)-induced colitis and DSS.^21^ The bile acid-FXR signaling pathway may represent an essential intervention target in NEC, and additional experiments are needed to decipher the role of FXR in NEC.

*B. fragilis*, a gram-negative anaerobic bacterium widely present on the mucosal surface of the human lower gastrointestinal tract, can be divided into enterotoxin-producing and non-enterotoxin-producing strains.^22^ Numerous studies have demonstrated that non-enterotoxin-producing *B. fragilis* possesses probiotic functions, including enhancing the host intestinal barrier, enriching gut microbiota, preventing intestinal pathogen infections, boosting intestinal immunity, and alleviating host inflammation of NEC.^23,24^ Therefore, non-enterotoxin-producing *B. fragilis is* considered one of the candidate strains for next-generation probiotics.^25^ Additionally, *B. fragilis* can influence several diseases, including intrahepatic cholestasis of pregnancy, by inhibiting FXR signaling via its BSH activity to modulate bile acid metabolism.^26^ However, whether *B. fragilis* influences FXR in NEC through BSH activity remains unclear.

In this study, we hypothesized that changes in bile acid profile may cause NEC. We propose that certain probiotics containing BSH can restore the balance of bile acid metabolism, activate the FXR-NLRP3 signaling pathway, repair intestinal damage, and alleviate NEC.

## Materials and methods

### Reagents and materials

Tryptone soy broth (TSB) and Man – Rogosa – Sharpe (MRS) medium were purchased from Huankai Microbial Sci. & Tech (Guangzhou, China). Standard samples of bile acids, including taurochenodeoxycholic acid (TCDCA), taurodeoxycholic acid (TDCA), and glycodeoxycholic acid (GDCA), were purchased from Sigma-Aldrich (MO, USA). Eosin Y Stain Solution and Modified Harris’ Hematoxylin Stain Solution were purchased from Solarbio (Beijing, China). The TRIzol reagent, a reverse transcription kit, and SYBR Green Master Mix were purchased from Takara Bio (Tokyo, Japan). A protein extraction kit was purchased from Beyotime Biotechnology (Shanghai, China). A bacterial genome extraction kit and TIANamp Stool DNA Kit were purchased from TianGen (Beijing, China). Glycine and taurine were purchased from Aladdin (Shanghai, China). Human IntestiCult™ Organoid Growth Medium and Gentle cell Dissociation Reagent were purchased from StemCell Technologies (Vancouver, Canada). GW4064, Guggulsterone E-Z, and Y-27632 were purchased from Selleck (Houston, TX, USA), MedChemExpress (Monmouth Junction, NJ, USA), and Abmole (Houston, TX, USA), respectively.

### Infant sample collection

Sample collection was approved by the Ethics Committee of Nanfang Hospital, China (NFEC-2021-430). Briefly, fecal samples were collected from 10 healthy premature infants and 9 infants with confirmed neonatal necrotizing enterocolitis (NEC). Surgically resected intestinal tissues were collected from infants with NEC. The diagnostic criteria for NEC followed the guidelines of IPGRP-2020CN004. None of the infants received bile acid binders (e.g., cholestyramine) or bile acid medications (e.g., ursodeoxycholic acid) during the study. None of the infants showed evidence of hepatic or gastrointestinal disorders, except NEC, and none had received antibiotics before fecal sample collection. Biosamples and patient clinical features (*n* = 19) were provided by the Department of Neonatology at Nanfang Hospital of Southern Medical University. Fresh fecal samples were collected within 2 h following defecation. Fecal samples were used to isolate bacterial strains, and the rest of the samples were stored at − 80°C until use. The clinical characteristics of the infants are presented in Table 1.

**Table 1.** Characteristics of neonates in necrotizing enterocolitis group and control group.

Characteristics	NEC (*n* = 9)	Control (*n* = 10)
Birth weight (g), mean ± SD	1012 ± 210	1222 ± 165
Gestational week (wk), mean ± SD	28.44 ± 2.01	28.80 ± 1.55
Premature, n (%)	9 (100)	10 (100)
Birth age (d), mean ± SD	24.11 ± 8.33	18.10 ± 7.03
TBA (μmol/L), mean ± SD	26.40 ± 9.30	14.87 ± 3.27

### Bile acids utilized for targeting metabolomics

Bile acid was extracted from infant and rat feces according to the manufacturer’s instructions with minor improvements. Briefly, 100 mg of frozen feces and 300 μL of PBS were placed in EP tubes and homogenized on ice. After centrifuging at 4°C and 14,000 × g for 15 min, the supernatant was collected. Then, 1 mL of antioxidant solution (0.2 mg/mL solution of BHT/EDTA in 1:1 MeOH:water) was added to 100 μL of homogenate and mixed by vortexing for 10 min, then centrifuged at 4°C and 14,000 rpm for 15 min. The supernatant was collected, evaporated to dryness under nitrogen, and resuspended in 50 μL of methanol-water (1:1, v/v). This was followed by vortexing for 5 min, and centrifuging at 4°C and 14,000 rpm for 15 min. The supernatant was filtered using a 0.22-um filter. As for the standard samples, each standard sample (deuterated bile acids, 250 nmol) was dissolved in antioxidant solution. The homogenized solution was then centrifuged, dried, resuspended in 50 μL of methanol-water, and filtered using a 0.22 um filter for LC-MS analysis (Q Exactive Focus Thermo Fisher Scientific/1290 Infinity series UHPLC System Agilent).

### Bacterial culture and cell culture

*Bacteroides fragilis* strain ATCC 25,285 (*B. fragilis*) was purchased from ATCC (Manassas, VA, USA) and cultured in TSB medium supplemented with 5% fetal bovine serum (GIBCO, Grand Island, NY, USA). *B. fragilis* was cultured anaerobically at 37°C for 24 h in an anaerobic cabinet (Mart, Drachten, The Netherlands). IEC-6 and HT-29 cells were purchased from ATCC (Manassas, VA, USA) and cultured in RPMI-1640 medium supplemented with 10% fetal bovine serum (GIBCO, Grand Island, NY, USA). Cells were cultured at 37°C with 5% CO_2_.

### Animal model

All animal studies were approved by the Institutional Animal Care and Use Committee of Southern Medical University. Two-week-old pregnant Sprague-Dawley (SD) rats (*n* = 15) were purchased from the Southern Medical University Laboratory Animal Center, China, and raised under specific pathogen-free conditions with free access to food and drinking water. All in vivo experiments were performed in accordance with the guidelines of our institution regarding the use of laboratory animals. Newborn SD rats (*n* = 80) were breastfed for three days after birth and randomly divided into 8 groups with no blinding. The NEC model was established in four-day-old rats using a previously reported protocol.^27^ Briefly, rat pups were stressed three times every day through a hypoxia treatment (breathing 5% oxygen + 95% nitrogen for 10 min), followed by cold stress (4°C for 5 min) for three days. All pups were fed LPS at 10 mg/kg dissolved in 0.1 mL sterile water via an intragastric tube once daily for three days to induce NEC. The rats received 500 mg/kg/day TCDCA or TDCA orally for three days. When treated with FXR agonists, the mice received 10 mg/kg Guggulsterone E-Z (E/Z-GS) orally for 3 days. The rats were euthanized with phenobarbital sodium and their organs and blood samples were collected. When the rats were treated with *B. fragilis*, they received 8 × 10^8^ CFU/ml, 200 µL/day orally for six days.

### Hematoxylin-eosin (HE) and immunohistochemical (IHC) staining

HE staining was performed according to the manufacturer’s instructions. An established scoring criterion^28^ was utilized to conduct the pathological injury assessment, with two independent pathologists blinded to the study. The criteria are as follows: 0 (normal) indicates no damage; 1 (mild) denotes slight submucosal and/or lamina propria separation; 2 (moderate) signifies moderate separation of submucosa and/or lamina propria, and/or edema in submucosal and muscular layers; 3 (severe) indicates severe separation of submucosa and/or lamina propria, and/or severe edema in submucosa and muscular layers, along with villous sloughing; and 4 (necrosis) represents the loss of villi and necrosis. Rats with a pathological injury score exceeding 2 were categorized as NEC.

For IHC staining, 5-mm thick paraffin-embedded sections were deparaffinized and the antigen was retrieved. Sections were blocked in 1% normal goat serum and H_2_O_2_ for 20 min and incubated overnight at 4°C with the rabbit anti-FXR polyclonal antibody, followed by incubation with the horse-radish peroxidase (HRP)-coupled secondary antibody at room temperature for 20 min. Staining intensity was evaluated using ImageJ software (V1.8.0, MD, USA). The IHC score ranges from 0 to 4, with 0 assigned to no positive staining (negative), 1 assigned to pale yellow (weak positive), 2 assigned to light brown (positive), and 3 assigned to dark brown (strong positive). The percentage of positive cells is graded on a scale of 0 to 4, with ≤ 25% scoring 1 point, 26%–50% scoring 2 points, 51%–75% scoring 3 points, and >75% scoring 4 points. The final score is obtained by multiplying the two scores together.

### RNA isolation and qRT-PCR

Total RNA were extracted from colon segments (0.1 g) and bacteria (0.1 g) using the TRIzol reagent. The RNA was converted to cDNA using a reverse transcription kit. Gene expressions were determined using qPCR SYBR Green Master Mix and a 7500 real-time quantitative PCR system (Applied Biosystems, Thermo Fisher Scientific, Waltham, MA, USA). Gapdh or 16S were used for normalization. Relative quantification of the target genes was performed using the 2^−ΔΔ^CT method. The primers used are presented in Table 2.

**Table 2.** Primers of qPCR.

Species	Gene symbol	Forward primer (5’-3’)	Reverse primer (5’-3’)
Rat	Il-6	GTCAACTCCATCTGCCCTTCAG	GGCAGTGGCTGTCAACAACAT
Rat	Gapdh	AGACAGCCGCATCTTCTTGT	TGATGGCAACAATGTCCACT
Bacteria	bsh	CACATATTGTGGCACGAACAATHGAR TGGGG	CTGTGCCCGGATACAGATTAACRTAR TTRTT
Bacteria	16S v3-v4	CCTACGGGNGGCWGCAG	GACTACHVGGGTATCTAATCC

### Infant intestinal organoids

Ileum intestinal organoids (normal portions of the intestine from NEC human infants after surgical resection, which were generously provided by the Department of Neonatology, Nanfang Hospital) were prepared according to the StemCell Technologies protocol. Briefly, intestinal tissue was cut into 1 mm^2^ pieces and digested with Gentle Cell Dissociation Reagent for 40 min at room temperature. Small-intestinal crypts were released by vigorous shaking and seeded onto Matrigel (BD Biosciences, San Jose, CA, USA) in a total volume of 50 µL to 24-well plates (NEST Biotechnology, Wuxi, China). To prevent anoikis, 10 µM Y-27632 was included in the Human IntestiCult™ Organoid Growth Medium for the first two days. Intestinal organoids were treated with 10 μg/mL LPS to establish the NEC model, as previously reported.^29,30^ In the treatment groups, organoids were treated with 1 × 10^8^ CFU of *B. fragilis*, 0.5 µM of TCDCA, and 0.5 µM of TDCA for one day. Subsequently, the organoids were cultured in Human IntestiCult™ Organoid Growth Medium for one day. The enteroid cell death was quantified using the Lactate Dehydrogenase (LDH) Assay Kit (Absin, China), following the experimental protocol outlined in the user manual. Briefly, the organoids in the dome were isolated following digestion, and cells were disrupted using bath sonication for 5 min. Protein concentration was measured, and then the samples were treated with working reagent and incubated at 37°C in the dark for 30 min. Absorbance at 450 nm was measured to quantify cell death.

### Gene set enrichment analysis (GSEA)

Raw data from GSE46619, GSE64801, and GSE198372 were downloaded from the GEO database of the NCBI. A robust multiarray averaging background correction, log2 transformation, and quantile normalization were performed on the raw expression data using the “Affy v1.62.0” and “limma v3.40.6” R packages.^31,32^ GSEA was performed on all three datasets using the software downloaded from www.broadinstitute.org/gsea.^33^ The latest molecular signatures database gene sets were used in the analysis, including “c2.cp.kegg.v7.5.1. symbols” and “MousePath_All_gmt-Format”. The number of permutations was set to 1,000.

### Co-immunoprecipitation (CO-IP) and western blotting

Total protein measurements were extracted as previously described,^34^ and 100 μg of the lysate was incubated overnight with mouse anti-FXR (1:200) monoclonal antibodies, and rabbit anti-NLRP3 (1:50) polyclonal antibodies, or IgG (as a negative control, 1:1000) at 4°C on a rocker platform. Then, the resulting complex was incubated with 20 µL of Protein A/G PLUS-Agarose for 5 h at 4°C. Complex beads were collected by centrifugation at 1,000 × g for 5 min at 4°C and washed four times with PBS. After the final wash, the supernatant was aspirated and discarded, and the remaining pellet was resuspended in 5× electrophoresis sample buffer. After boiling the samples for 3 min, aliquots were prepared and analyzed via western blotting.

Rat ileum tissues and liver tissues were lysed with RIPA buffer containing a protease inhibitor cocktail and a phosphatase inhibitor cocktail (CWBIO). The following primary antibodies were used for western blot analysis: mouse anti-FXR/NR1H4 monoclonal antibody (72105S, CST), rabbit anti-FGF19 polyclonal antibody (DF2651, Affinity), rabbit anti-OATP polyclonal antibody (A8452, Abclonal), rabbit anti-NTCP polyclonal antibody (A12721, Abclonal), mouse anti-CYP7A1 monoclonal antibody (A22897, Abclonal), mouse anti-BSEP monoclonal antibody (67512–1–Ig, Proteintech), rabbit anti-NLPP3 polyclonal antibody (19771–1–AP, Proteintech). The following secondary antibodies were used for western blot analysis: HRP-conjugated Goat anti-Rabbit IgG (H+L) (AS014, Abclonal) and HRP-conjugated Goat anti-Mouse IgG (H+L) (AS080, Abclonal).

### Taxonomic characterization of the bsh gene

Reference genomes of 1,520 cultivated human gut bacteria were obtained from the NCBI database (No. PRJNA48274833).^35^ The de novo assembly was performed as previously described.^36^ The genes and related proteins in these bacterial genomes were predicted using Prokka (v1.3), and taxonomic information regarding these genes and proteins was directly extracted from the strain names.^37^ Pairwise amino acid sequence alignments and multiple alignments of *bsh* sequences were performed using ClustalW in the MEGA software (v11.0).^38^ A phylogenetic tree was constructed using the maximum likelihood method in MEGA with 1,000 bootstrap replications. This process was conducted using the interactive tree of life (iTOL) (v5.0, https://itol.embl.de).

### bsh gene detection and BSH activity assay

Bile salt hydrolase (*bsh*) gene detection and BSH activity analyses were performed as previously described.^39^

First, every five strains of bacteria (213 probiotics and *B. fragilis*) were mixed to extract DNA using a bacterial genome extraction kit, and the *bsh* gene was detected using PCR. The primers used for *bsh* are listed in Table 2. When PCR products were detected, the DNA of the five strains was extracted and analyzed.

Then, 2 × 10^9^ CFU of bacteria with the *bsh* gene were washed and suspended twice with PBS, and then broken with an ultrasonic cell crusher for 30 min (2:3,500w). The 10 µL suspensions were spot inoculated on a dry surface of MRS agar supplemented with 5 g/L TDCA or 5 mM GDCA and incubated at 37°C for 72 h. Positive BSH activity was recorded when a white precipitate or halo was produced and the reaction with ninhydrin was purple.

Finally, 2 × 10^9^ CFU of bacteria with the *bsh* gene were washed and suspended twice in PBS and then broken with an ultrasonic cell crusher for 3 min. The mixture was centrifuged at 4°C and 10,000×g for 4 min. The 100 µL supernatant was added to 1.8 mL PBS, and 100 µL conjugated bile salt (200 mM) and incubated at 37°C for 30 min. The reaction (500 µL) was terminated with 500 µL of 15% trichloroacetic acid (w/v). The mixture was centrifuged at 4°C 14000 × g for 10 min. Then, the 100 µL supernatant was mixed with 1.9 mL of ninhydrin chromogenic solution and bathed in boiling water for 14 min. The absorption at 570 nm was measured after 3 min of cooling. Standard curves were constructed using glycine (37.5 mg/L) and taurine (62.5 mg/L). The BSH total activity (μmol(min×mL)-1) was defined as follows:

Total activity = 4/3 × Concentration of amino acids.

### Knockout of bsh using CRISPR/Cas9

The *bsh* gene in *B. fragilis* was knocked out using CRISPR/Cas9 according to a previously published protocol.^40^ Briefly, sgRNA (5’- TTCAGTTGGTATCATATGTGCTGGC-3’) targeting *bsh* in *B. fragilis* was designed using the online tool CHOPCHOP (https://chopchop.cbu.uib.no/). A total of 1998 bp of homology arms (999 bp upstream and 999 bp downstream of *bsh*) and 25 bp of sgRNA were inserted into pB025 using Gibson assembly. The plasmid was transformed into competent *E. coli* S17–1 cells and selected for growth on LB plates containing 100 µg/mL ampicillin. After overnight incubation at 37°C, colonies were picked to confirm the correct insertion of sgRNA expression cassettes and homologous arms. Donor and recipient strains were combined at a 1:5 ratio (donor: recipient, *E. coli* S17–1: *B.* *fragilis*) in a culture volume ratio (200:1000 μL), centrifuged at 5000 g for 15–20 min, resuspended in 100 μL of BHI liquid medium, and plated as dime-sized puddles on nonselective BHI-blood agar plates for 24 h at 37°C under aerobic conditions to allow for conjugation. Once conjugation was complete, mating lawns were shaved and resuspended in 1 mL BHI. After a 1:100 dilution of the bacteria, 150 μL of bacteria were spread onto plates with antibiotics (Gen, Erm), respectively. After culturing the plates at 37°C for two days, colonies were picked for sequencing to confirm whether the *bsh* gene was knocked out.

### 16S rRNA sequencing

Total genomic DNA was extracted using the TIANamp Stool DNA Kit. The V3-V4 region of the 16S rRNA gene was amplified using 338F (5’-ACTCCTACGGGAGGCAGCAG-3’) and 806 R (5’-GGACTACHVGGGTWTCTAAT-3’) primers. Samples were sequenced on an Illumina HiSeq 2500 platform (Illumina, CA, USA) using MAGIGENE (Guangzhou, China). Paired-end reads were merged using the fast length adjustment of short reads, and sequence analysis was performed using UPARSE.^41^ Sequences with ≥ 97% similarity were assigned to the same operational OTUs. The diversity and composition of the bacterial communities and the linear discriminant analysis effect size (LEfSe) were performed using the website ImageGP (http://www.ehbio.com/ImageGP/).^42^

### Enzyme-linked immunosorbent assay (ELISA)

A total of 100 mg of ileal tissue was homogenized in 100 μL of PBS. The homogenate was centrifuged at 14,000 × g, 4°C for 15 min, and the supernatant was collected for subsequent ELISA. The concentration of IL-1β from the ileum was detected using an ELISA kit according to the manufacturer’s instructions (ERC007.96, Neobioscience).

### Statistical analysis

All data are expressed as mean ± standard deviation and statistical significance was set at *p* ≤ 0.05. Statistical differences between the experimental groups were analyzed using one-way ANOVA and Student’s t-test, and all statistical analyses were performed using SPSS (version 22.0; IBM, NY, USA). All experiments were performed in triplicate.

## Results

### Disorder of bile acid metabolism in infants with NEC

Previously, we found that the gut microbiomes of healthy and NEC-affected infants were significantly different.^43,44^ Because bile acid metabolism is closely related to the gut microbiome, we wondered whether bile acid metabolism differs between healthy infants and infants with NEC. Therefore, the serum and feces of 10 healthy infants and 9 infants with NEC (gestational age ≤32 weeks and birth weight ≤1500 g) were collected. The level of serum total bile acid (TBA) in NEC-affected premature infants (26.40 ± 9.30 μmol/L) was significantly higher than the normal range (14.87 ± 3.27), as is shown in Table 1.

**Figure 1.**
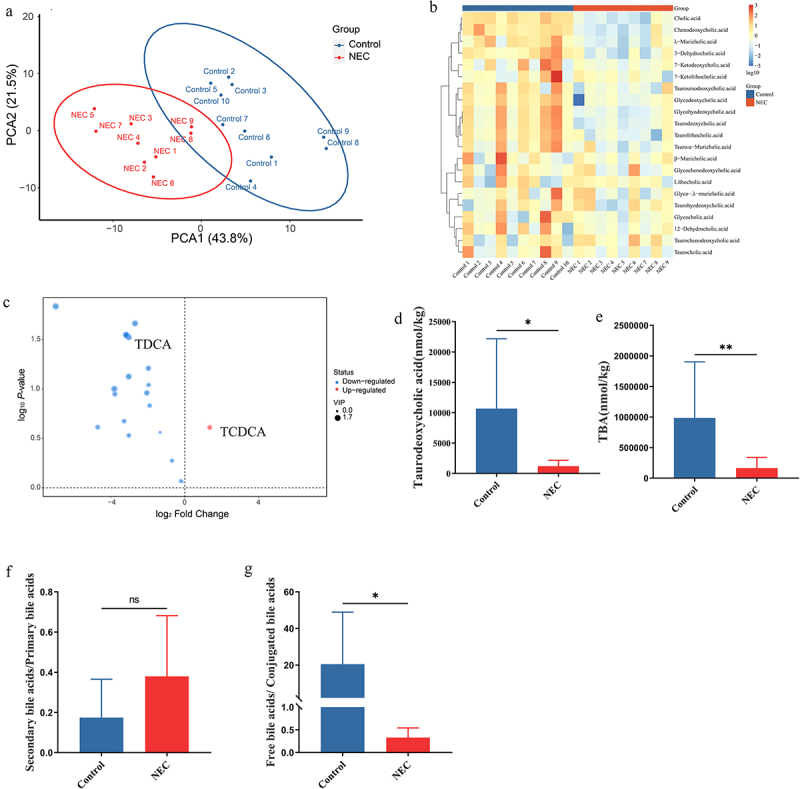
Differences in bile acid levels in feces between NEC patients (*n* = 9) and healthy infants (*n* = 10). (a). PCA of bile acids. (b). The heatmap displays concentrations of bile acids, and the data was transformed with Log10. (c). Volcano plot of differential bile acids. (d). Concentration of TDCA in feces of healthy infants and infants with NEC. (e). Concentration of TBA in the feces of healthy infants and infants with NEC. (f). The ratio of secondary bile acids/primary bile acids. (g). The ratio of free bile acids/conjugated bile acids. *p < 0.05; **p < 0.01.

To further explore the differences in bile acid levels in feces between NEC patients and healthy infants, we conducted targeted metabolomics using LC-MS to assess bile acid composition. According to the principal components analysis (PCA) of bile acids in feces, the bile acid composition of infants with NEC was significantly different from that of healthy infants (Figure 1(a)). Based on the heatmap of the NEC and control groups (Figure 1(b)), primary bile acids, including TCDCA, significantly increased, whereas the secondary bile acids of TDCA significantly decreased in the NEC group (Figure 1(c-d)). The most striking difference in bile acid profiles was that the TBA of feces was significantly lower in infants with NEC than in healthy infants (Figure 1(e)). No significant difference was observed in the ratio of secondary/primary bile acids and TUDCA between healthy infants and infants with NEC (Figure 1(f) and Supplementary Figure S1). However, in the infants with NEC, the ratio of unconjugated/conjugated bile acids was significantly lower than that in the healthy infants (Figure 1(g)). Serum TBA levels increased and fecal TBA levels decreased in infants with NEC. These results indicated a disorder in bile acid metabolism in infants with NEC.

### TCDCA aggravates the pathogenesis of NEC and TDCA alleviates NEC in vivo and in vitro

**Figure 2.**
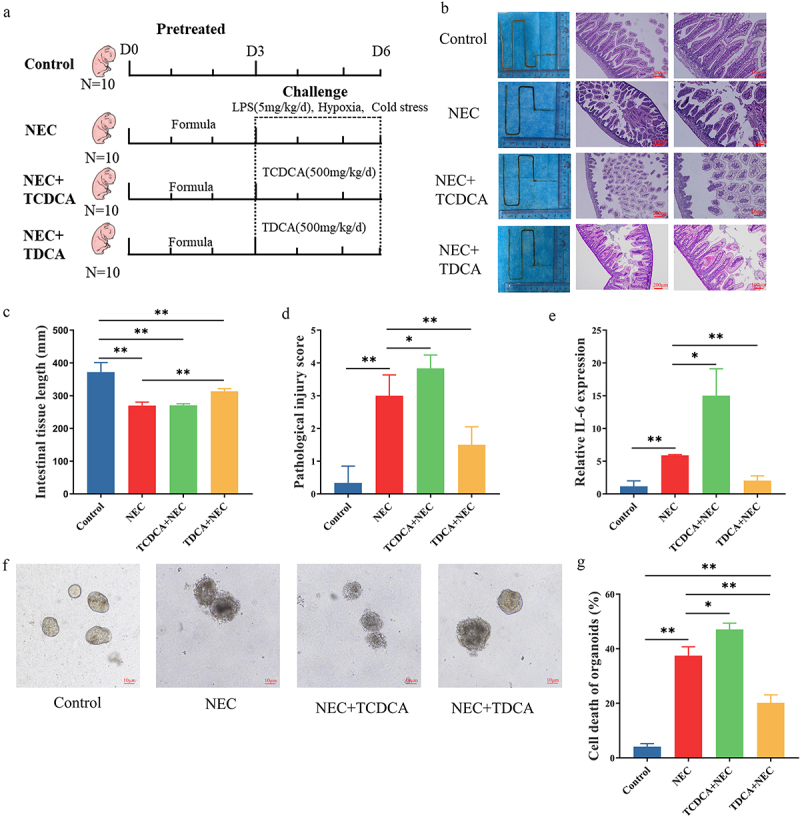
TCDCA promoted NEC pathogenesis. (a). The grouping and flowchart of the rat experiment (*n* = 10 per group). (b). Images of the small intestine in different groups and HE staining of the rat ileum. (c). The length of the small intestine in different groups. (d). Inflammatory scores of HE staining in different groups. (e). Relative mRNA expression of Il-6 in the ileum. (f). Images of ileum organoids obtained under a light microscope. (g). Cell death in the ileum organoids in each group (*n* = 30 per group). *p < 0.05; **p < 0.01.

Because TCDCA was the most significantly elevated bile acid, we investigated whether it promoted or inhibited NEC pathogenesis. We established an animal model of NEC and treated the newborn rats with TCDCA or TDCA (Figure 2(a)). Rats in the NEC group had short and small intestines with severe intestinal damage, including disruption of the intestinal epithelium, coagulative necrosis of parts of the ileum, and dense inflammatory cell infiltration (Figure 2(b-d)). After being orally treated with TCDCA, the rats in the NEC+TCDCA group had shorter intestine tissues and more severe damage than the NEC group. TCDCA aggravated the loss of villi and necrosis, which was observed in the NEC group (Figure 2(b-d)). Moreover, the mRNA levels of the proinflammatory cytokine Il-6 in the NEC group were significantly higher than those in the control group, whereas the NEC + TCDCA group exhibited a higher level of Il-6 than the NEC group (Figure 2(e)). However, after being orally treated with TDCA, the rats in the NEC+TDCA group had longer intestine tissues, lower HE scores of inflammation, and less damage than the NEC group (Figure 2(b-d)). The mRNA levels of Il-6 in the TDCA+NEC group were significantly lower than those in the NEC group (Figure 2(e)).

Intestinal organoids were also used to evaluate the role of TCDCA and TDCA in NEC. In the NEC group, the structural integrity of the organoids was destroyed, and dead cells were observed in the core of the organoids. This damage and cell death were more severe in the NEC + TCDCA group than in the NEC group (Figure 2(f-g)). The damage and cell deaths in the TDCA+NEC group were less than those in the NEC group (Figure 2(f-g)). These findings indicate that TCDCA aggravated the pathogenesis of NEC and TDCA alleviated the NEC.

### FXR and other bile acid metabolism-related genes were abnormal in NEC

**Figure 3.**
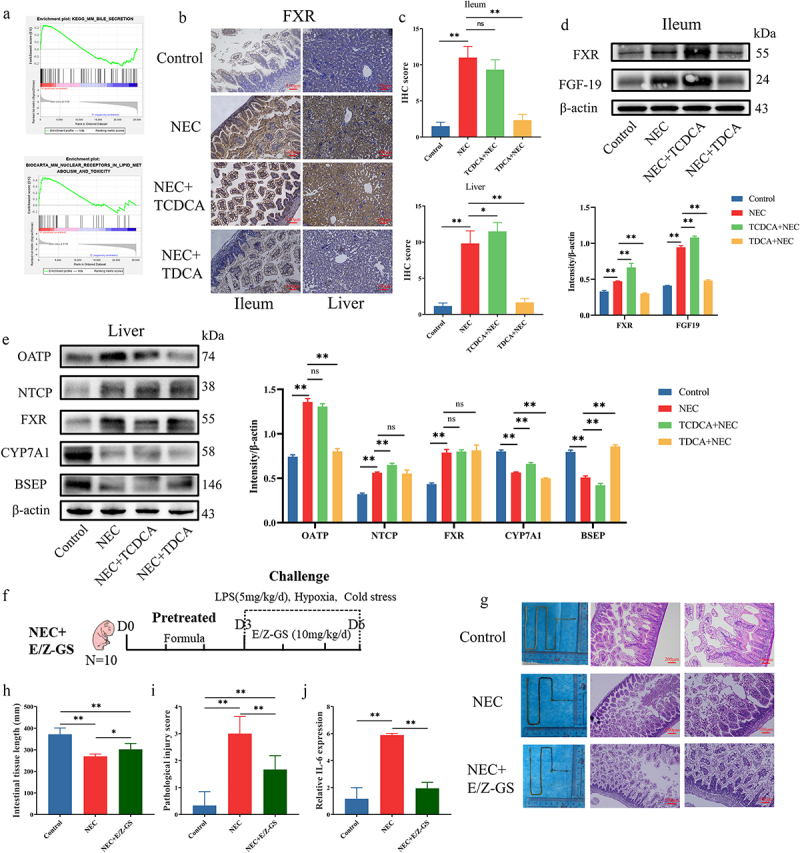
Bile acid metabolism was disorderly in NEC. (a). Enrichment plot of KEGG pathways using GSEA. (b). IHC staining of FXR in the ileum and liver. (c). IHC staining scores of FXR in the ileum and liver. (d). The protein levels of FXR and FGF19 in the ileum and comparison of grayscale values of Western blot bands. (e). The protein levels of FXR, OATP, NTCP, CYP7A1, and BSEP in the liver and comparison of grayscale values of Western blot bands. (f). The grouping and flowchart of the rat experiment (*n* = 10 per group). (g). Images of the small intestine in different groups and HE staining of the rat ileum. (h). The length of the small intestine in different groups. (i). Inflammatory scores of HE staining in different groups. (j). Relative mRNA expression of Il-6 in the ileum. *p < 0.05; **p < 0.01.

After conducting the GSEA in GSE198372, an expression array of the mice NEC model, we observed that “KEGG_MM_BILE_SECRETION” and “BIOCARTA_MM_NUCLEAR_RECEPTORS_IN_LIPID_METABOLISM_AND_TOXICITY” were enriched in NEC groups (Figure 3(a)). Because the FXR was involved in those two pathways and was also known as the classical receptor of TCDCA, we detected the expression of FXR in the ileum and liver using IHC. IHC staining indicated that the protein level of FXR was significantly higher in the NEC group than in the control group, both in the ileum and liver. Following treatment with TCDCA, the FXR expression was higher than in the Control group (Figure 3(b-c)). Upon treatment with TDCA, the FXR expression was lower than that in the NEC group (Figures 3(b-c)).

We further measured the protein levels of FXR-regulated genes in the ileum and liver using western blot analysis. The protein level of FXR is activated in the ileum and liver of the NEC group and TCDCA+NEC group, while FXR is decreased after treatment with TDCA (Figure 3(d)). The changes in the FGF19 protein levels in the ileum, which is downstream of FXR, were consistent with those of FXR (Figure 3(d)). The changes in OATP and NTCP protein levels in the liver were consistent with those in FXR (Figure 3(e)). The changes in CYP7A1 and BSEP protein levels in the liver exhibited an opposite trend to that of FXR (Figure 3(e)).

We established an animal model of NEC and treated the newborn rats with FXR inhibitor E/Z-GS (Figure 3(f)). The reduction in intestine length, HE scores of inflammation, and ileum damage were reversed following the E/Z-GS treatment (Figure 3(g-i)). The mRNA level of Il6 in the ileum was reversed with the E/Z-GS treatment (Figure 3(j)).

Collectively, these observations show that FXR and bile acid metabolism-related genes were abnormal in NEC and that FXR participated in the pathogenic mechanism of NEC.

### FXR interacts with NLRP3 and regulates protein degradation

**Figure 4.**
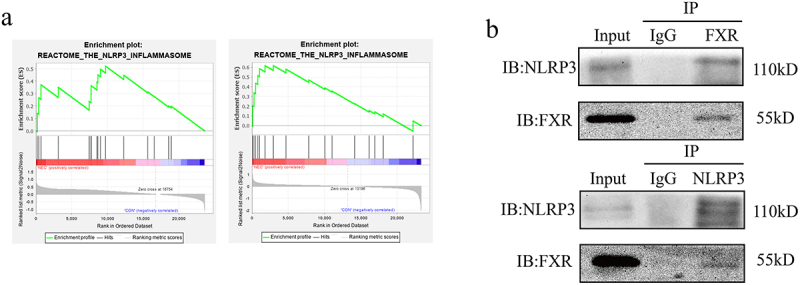
NLRP3 interacted with FXR. (a). Enrichment plot of KEGG pathways in the GSE46619 and GSE64801. (b). Co-IP identified the interaction between NLRP3 and FXR.

After conducting the GSEA in GSE46619 and GSE64801, the two expression arrays of intestinal tissues from infants with and without NEC, we observed that “REACTOME_THE_NLRP3_INFLAMMASOME” and the other seven pathways were enriched in NEC groups (Figure 4(a)). Because FXR reportedly interacted with NLRP3 in cholestasis-associated sepsis,^45^ we investigated whether FXR has the same function in NEC. COIP was performed to determine whether FXR interacts with NLRP3. Western blotting revealed an interaction between FXR and NLRP3 in IEC-6 cells (Figure 4(b)). These results suggested that FXR interacted with NLRP3 to regulate its degradation.

### B. Fragilis *contains bile salt hydrolase (BSH)*

**Figure 5.**
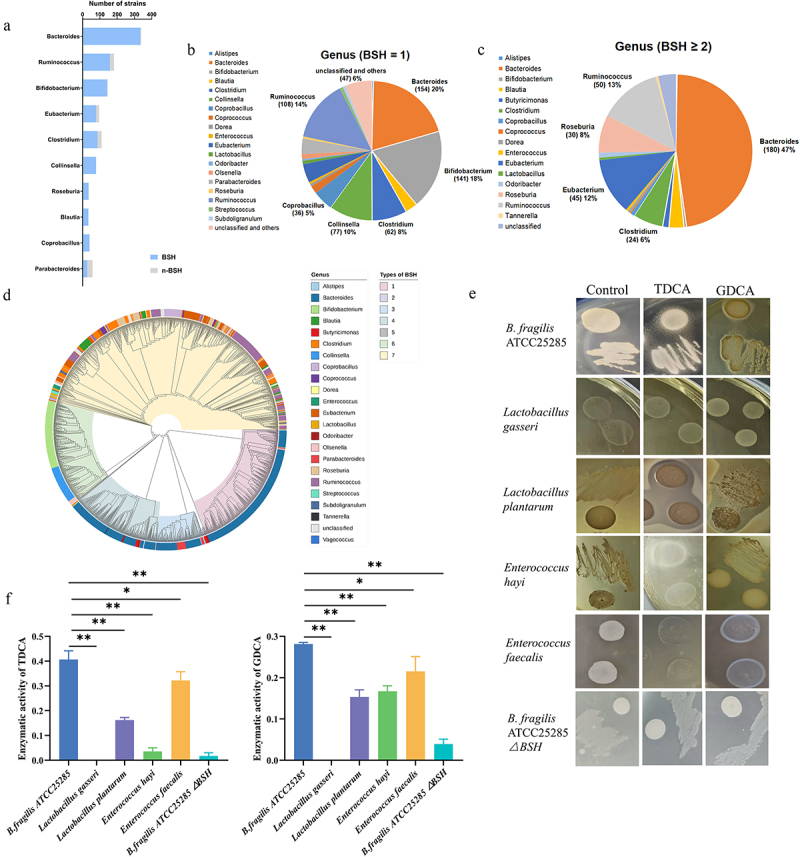
*bsh* in bacteria strains and *B. fragilis* had efficient BSH enzyme activity. (a). Number of strains in top 10 genera with *bsh*. (b). Pie chart of strains with one *bsh* gene. (c). Pie chart of strains with two or three paralogous *bsh* genes. (d). Phylogenetic analysis of 1577 *bsh* genes. (e). Enzyme activity of BSH in five probiotics and *B. fragilis* knockout *bsh* strain. (f). Determining the amount of amino acids liberated from TDCA and GDCA to show the enzyme activity of BSH. *p < 0.05; **p < 0.01.

TCDCA was closely related to NEC and activated FXR in vivo, which indicated that reducing the concentration of this compound may represent a novel treatment. Several studies have shown that BSH converts TCDCA to CDCA in bacteria. Therefore, we decided to identify bacteria with high BSH activity, especially probiotics, to ensure their biological safety. First, we performed a bioinformatics analysis of *bsh* using 1,520 reference genomes from cultivated human gut bacteria (CHGB). Bacteria were assigned to 29 genera, including *Bacteroides, Ruminococcus, Bifidobacterium*, *Eubacterium*, and *Clostridium* from three phyla (Figure 5(a)). Nearly 98.5% of the *Bacteroides* strains harbored *bsh* genes. Furthermore, 20% (154 strains) of the *Bacteroides* strains encoded only one *bsh* gene (Figure 5(b)), whereas 47% (180 strains) of *Bacteroides* and 13% (50 strains) of *Ruminococcus* strains had two or three paralogous *bsh* genes. This suggests that *Bacteroidetes* strains have an elevated BSH activity rate (Figure 5(c)). Distinguishing BSH by genera may not represent a rational method because BSH paralogs are harbored in the bacterial genome. Therefore, the 1579 *bsh* genes in the CHGB were classified into seven phylotypes via phylogenetic analysis, with the number of sequences stated in parenthesis (Figure 5(d)). Collectively, we predicted that Bacteroidetes strains would demonstrate a high BSH activity rate. We conducted BSH activity experiments to determine the most efficient probiotic. We systematically detected the *bsh* genes in 214 bacterial strains. qPCR showed that *B. fragilis*, *Lactobacillus gasseri*, *Lactobacillus plantarum*, *Enterococcus hayi*, and *Enterococcus faecalis* possessed BSH-encoding genes. The four probiotic strains also exhibited BSH enzyme activity (Figure 5(e)). The calculated total BSH activity in *B. fragilis* was significantly higher than those of the other bacteria (Figure 5(f)). After knocking out one of the *bsh* genes in *B. fragilis*, the BSH enzyme activity significantly reduced (Figure 5(e)). The calculated total BSH activity in *B. fragilis*△BSH strain was significantly lower than those in *B. fragilis* (Figure 5(f)). The mRNA levels of *bsh* genes in four probiotic strains, *B. fragilis* and *B. fragilis*△BSH strain were determined and found to constitute BSH activity (Supplementary Figure S2). These findings suggest that *B. fragilis* contained the *bsh* gene and demonstrated BSH activity based on bioinformatic prediction and experimental verification.

### B. Fragilis *alleviates NEC in vivo*

**Figure 6.**
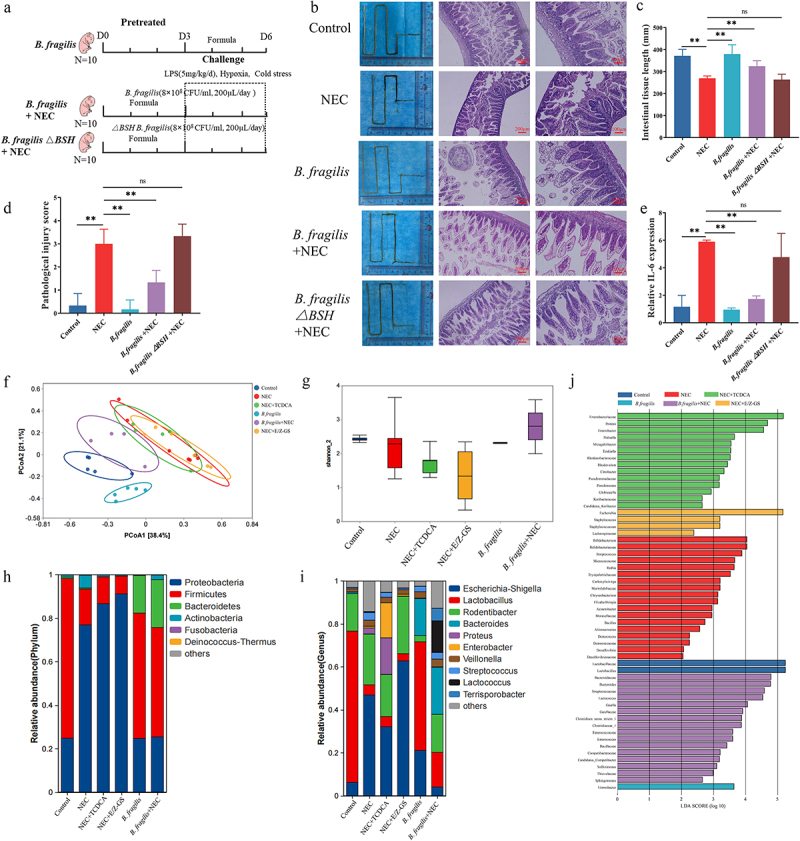
The therapeutic effects of *B. fragilis* in the NEC model. (a). The grouping and flowchart of the rat experiment (*n* = 10 per group). (b). Images of the small intestine in different groups and HE staining of the rat ileum. (c). The length of the small intestine in different groups. (d). Inflammatory scores of HE staining in different groups. (e). Relative mRNA expression of Il-6 in the ileum. (f). PcoA of the gut microbiota in different groups. (g). Shannon index of the gut microbiota. (h). Relative abundance of microbiota at the phylum level. (i). Relative abundance of microbiota at the genus level. (j). Lefse analysis of microbiota, with LDA score ≥ 2. *p < 0.05; **p < 0.01.

We evaluated the therapeutic effects of *B. fragilis* and the *bsh* gene in the NEC model (Figure 6(a)). Following intervention with *B. fragilis*, the short intestines of the NEC model improved (Figure 6(b-c)). HE staining also showed that the HE scores of inflammation and intestinal damage of NEC rats were alleviated in the NEC + *B. fragilis* group (Figure 6(b-d)). However, *B. fragilis*△BSH strain failed to improve the short intestines, inflammation, and intestinal damage of NEC (Figure 6(b-d)). The transcription level of Il-6 in NEC rats was also reduced following the *B. fragilis* treatment with the presence of the *bsh* gene (Figure 6(e)), thereby indicating that *B. fragilis* effectively relieved NEC enteritis in newborn rats via the *bsh* gene.

Next, we investigated whether *B. fragilis* modulates the composition of the microbial community to exert a protective effect against NEC. Principal Coordinate Analysis (PCoA) of the gut microbiota revealed a distinct separation between the *B. fragilis*+NEC group and the NEC group. Notably, the *B. fragilis*+NEC group exhibited a closer resemblance to the Control group, signifying a significant influence of oral *B. fragilis* treatment. Interestingly, the NEC group, NEC+TCDCA group, and NEC+E/Z-GS group clustered together, implying a minimal impact of TCDCA and E/Z-GS on the intestinal microbiota (Figure 6(f)). Based on α diversity estimates, the decreased Shannon index of the NEC, NEC+TCDCA, and NEC+E/Z-GS group was reversed by *B. fragilis* (Figure 6(g)). At the phylum level, the decreased abundance of Firmicutes and the increased abundance of Proteobacteria were reversed following the *B. fragilis* treatment (Figure 6(h)), and at the genus level, the decreased abundance of *Lactobacillus* and increased abundance of *Escherichia-Shigella* were also reversed after *B. fragilis* treatment, thus indicating that *B. fragilis* restores gut microbiota dysbiosis in NEC (Figure 6(i)). Lefse analysis revealed that pathogenic bacteria, such as *Streptococcus*, *Staphylococcus*, and *Escherichia coli*, exhibited increased abundance in the NEC, NEC+TCDCA, and NEC+E/Z-GS groups. In contrast, in the *B. fragilis*+NEC group, the abundance of *Bacteroides* and *Lactococcus* showed significant differences, suggesting that *B. fragilis* can colonize the gut and rectify gut microbiota dysbiosis (Figure 6(j)).

### B. Fragilis *alleviates NEC by regulating bile acid metabolism and reducing FXR-NLRP3 signaling*

**Figure 7.**
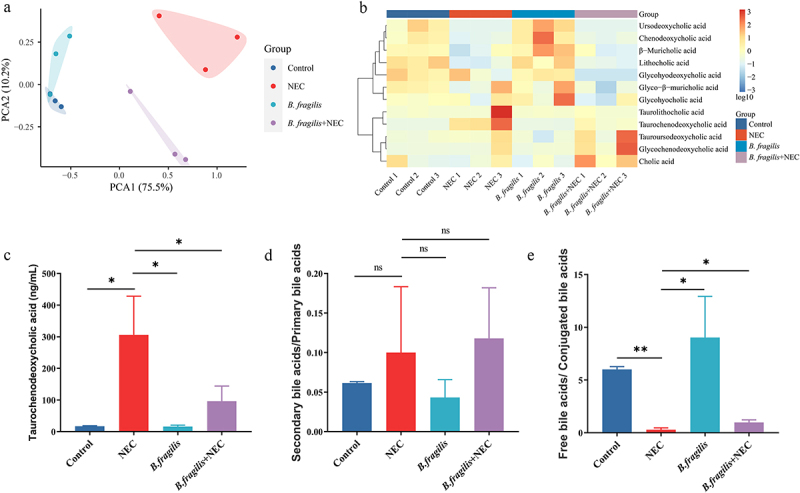
The effects of *B. fragilis* on bile acid metabolism (*n* = 3 per group). (a). PCA of the bile acids in different groups. (b). The heatmap displays concentrations of bile acids, and the data was transformed with Log_10_. (c). Concentration of TCDCA in feces of rats in different groups. (d). The ratio of secondary bile acids/primary bile acids. (e). The ratio of free bile acids/conjugated bile acids. *p < 0.05; **p < 0.01.

We also explored whether *B. fragilis* alleviates NEC by regulating bile acid metabolism. The PCoA of the bile acids revealed a distinct separation between the *B. fragilis*+NEC group and the NEC group (Figure 7(a)). The concentration of TCDCA in the NEC group significantly increased, compared with the Control group and *B. fragilis*+NEC group (Figures 7(b-c)). The ratio of secondary/primary bile acids among the groups did not exhibit statistically significant changes (Figure 7(d)). The ratio of unconjugated/conjugated bile acids in the NEC group was significantly lower than those in the Control and *B. fragilis*+NEC groups (Figure 7(e)).

**Figure 8.**
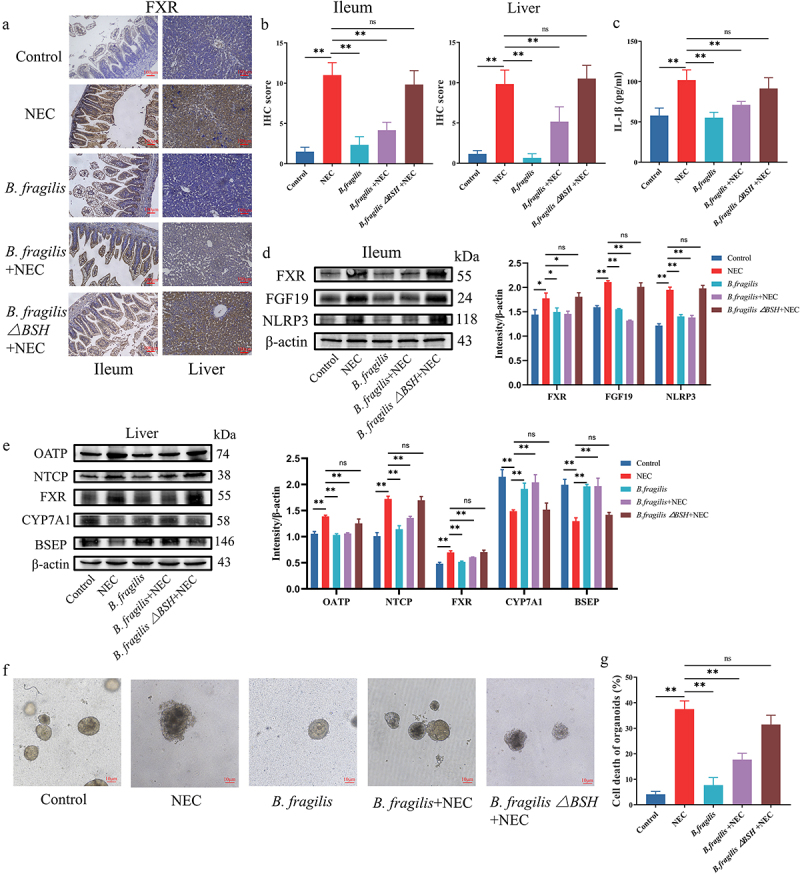
*B. fragilis* inhibited the FXR-NLRP3 signaling pathway and alleviated NEC. (a). IHC staining of FXR in the ileum and liver. (b). IHC staining scores of FXR in the ileum and liver. (c). The concentration of IL-1β in the ileum. (d). The protein levels of FXR and FGF19 in the ileum and comparison of grayscale values of Western blot bands. (e). The protein levels of FXR, OATP, NTCP, CYP7A1, and BSEP in the liver and comparison of grayscale values of Western blot bands. (f). Images of ileum organoids taken under a light microscope. (g). Cell death in the ileum organoids in each group (*n* = 30 per group). *p < 0.05; **p < 0.01.

Then, we explored whether *B. fragilis* and its *bsh* gene inhibited the FXR-NLRP3 signaling pathway. IHC staining was performed to detect FXR expressions in the ileal and liver tissues. The results showed that *B. fragilis* inhibited increased FXR expression in NEC (Figure 8(a-b)). Treatment with *B. fragilis*△BSH strain failed to inhibit the FXR expression in the ileum and liver (Figure 8(a-b)). We conducted western blotting and ELISA to detect the protein level of genes in FXR-NLRP3 signaling (Figure 8(c-d)). The results showed that the protein level of FXR is activated in the ileum and liver of the NEC group, and FXR is decreased after treatment with *B. fragilis* (Figure 8(d)). The changes in FGF19, NLRP3, and IL-1β in the ileum, which is downstream of FXR, were consistent with FXR (Figure 8(c-d)). The changes in OATP and NTCP protein levels in the liver were consistent with those of FXR (Figure 8(e)). The changes in CYP7A1 and BSEP protein levels in the liver exhibited an opposite trend to FXR (Figure 8(e)). However, those changes were reversed after the knockout of the *bsh* gene in *B. fragilis*.

Finally, we evaluated the in vitro therapeutic effects of *B. fragilis* and BSH. The damaged structural integrity and cell death of the organoids in the NEC group were reversed through the treatment with *B. fragilis* (Figure 8(f-g)). The damage and cell death of organoids were increased after the knockout of *bsh* in *B. fragilis* (Figure 8(f-g)). These findings suggest that *B. fragilis* alleviated NEC by restoring gut microbiota dysbiosis, regulating bile acid metabolism, and inhibiting the FXR-NLRP3 signaling pathway.

## Discussion

In this study, we identified a disorder in bile acid metabolism in NEC and a toxic effect of TCDCA on the progression of NEC. We used *B. fragilis* to treat NEC in newborn rats and human intestinal organoids. *B. fragilis* effectively restored the balance of the gut microbiota and bile acid metabolism, thereby alleviating ileal damage. Mechanistically, *B. fragilis* contained a BSH enzyme that converted toxic TCDCA to nontoxic bile acids and inhibited the downstream FXR-NLRP3 pathway. This study establishes *B. fragilis* as an effective and safe therapeutic agent to protect premature infants from NEC.

In our study, TBA levels were significantly higher in the blood of infants with NEC after they were provided a diagnosis than in healthy premature infants. Moreover, TBA and secondary bile acids in the feces of infants with NEC were significantly lower than those in healthy premature infants, whereas primary bile acids, especially TCDCA, were upregulated. In addition, TCDCA accelerated NEC pathogenesis. These results indicate abnormal bile acid metabolism in infants with NEC. This finding is consistent with that of previous research on neonatal rats, which also found decreased removal of ileal BAs.^46^ However, the intestinal bile salts of feces from 10 infants with NEC and 20 healthy infants showed opposite results; the total unconjugated BAs were threefold higher in infants with NEC than in healthy infants before the development of NEC.^47^ This contradiction can be explained by the different time points of fecal collection. In addition, previous analysis of fecal samples from infants with NEC revealed significant variabilities among individuals and found no notable distinction of TCDCA between infants with NEC and healthy infants.^48^ The disparities in this conclusion may stem from sample size limitations.

We observed that TDCA reduced the NEC severity, whereas TCDCA alleviated NEC, which contradicts a previous publication indicating that DCA exacerbates NEC.^15^ A recent seminal study has revealed that gut microbiota can modify bile acids and uncover various novel bacterial-derived bile acids that evade detection by conventional LC-MS methods.^49^ These novel bile acids serve significant physiological functions, implying numerous undiscovered relationships between gut microbiota and bile. The mitigation of NEC by TDCA in our study may be associated with the gut microbiota composition in our rat model. Additionally, TDCA exhibits greater hydrophobicity than TCDCA, and several studies have linked this property to increased cytotoxicity.^50–52^ Our findings, along with previous research,^47^ have identified reduced levels of free bile acids and conjugated bile acids in fecal samples from patients with NEC. Free bile acids demonstrate higher hydrophobicity and greater cytotoxicity. Therefore, we propose that the hydrophobicity-induced cytotoxicity of TDCA or TCDCA may not be the primary influencing factor in NEC.

After assessing the protein levels of bile acid metabolism-related genes, the expression of FXR and FGF19 increased in the ileum. Moreover, the protein levels of FXR, NTCP, OATP increased, and the level of CYP7A1 and BSEP decreased in the liver. Western blot analysis results indicated that during NEC, the synthesis of intrahepatic bile acids significantly increased and the excretion of intestinal bile acids was reduced, resulting in the accumulation of TBA in the blood. This finding is consistent with previous research showing that intestinal FXR and Asbt were increased in NEC animal models.^53,54^ Changes in FGF19, OATP, and BSEP expression have not yet been reported. We also observed increased levels of FXR, Ntcp, and Oatp in the liver along with decreased levels of Bsep. This observation contrasts with previous studies indicating that FXR negatively regulates the expression of Ntcp and Oatp, while positively regulating Bsep in the liver.^55,56^ This discrepancy may be attributed to the nuclear-cytoplasmic shuttling and post-translational modifications of FXR, warranting further investigation.

Promising therapies for NEC are broadly divided into probiotics, small molecules, and biologic agents.^57^ The use of probiotics to treat NEC has been verified in several large clinical trials.^58^ A systematic review of 56 trials in 10,812 infants showed that supplementation with *Bifidobacterium* spp., *Lactobacillus* spp., *Saccharomyces* spp., and *Streptococcus* spp., alone or in combination, reduced mortality and late-onset invasive infection.^59^ In the current study, treatment with *B. fragilis* significantly alleviated intestinal damage in neonatal NEC rats. *B. fragilis* has reportedly demonstrated therapeutic effects in several types of inflammatory enteritis, including *Cronobacter sakazakii*-induced NEC, dextran sodium sulfate-induced inflammatory bowel disease and ulcerative colitis.^24,60,61^

One of the underlying mechanisms of the *B. fragilis*-mediated alleviation of NEC is that the bacteria contains BSH, which regulates bile acid metabolism. Bioinformatics analyses of *bsh* in the gut microbiota have indicated that most of the strains in Bacteroides and Bifidobacterium contain BSH, which is consistent with our results.^62,63^ However, in our study, we mainly focused on the *bsh* gene of cultured bacteria from the human gut, and the culturability of bacteria is a critical foundation for the development of probiotics. We also used three types of experiments to validate the *bsh* gene and BSH activity in strains to avoid false positives in the bioinformatic analysis. The *B. fragilis* treatment also regulated the protein levels of bile acid metabolism-related genes, which resulted in increased bile acid transport in the liver, reduced reabsorption of bile acids in the intestine, and promoted bile acid excretion in feces. However, the role of *B. fragilis* in regulating bile acid metabolism in neonatal rats has not yet been elucidated.

Another new finding regarding the mechanism by which *B. fragilis* alleviates NEC is the inhibition of the activated FXR-NLRP3 signaling pathway. FXR interacted with NLRP3 and inhibited its protein degradation. It has been reported that NLRP3 activation promotes acute intestinal injury in NEC and the blockage of NLRP3 ameliorates injury.^64^ However, some studies have demonstrated that FXR negatively regulates the NLRP3 inflammasome in cholestasis-associated sepsis and reticulum stress-induced liver injury.^45,65^ The role of FXR in regulating the NLRP3 inflammasome is contradictory, and a potential reason for this discrepancy is that different types of disease models have been used.

This study has three limitations. First, the exact binding sites of FXR and NLRP3 are unknown, and further experiments are needed for validation. Thus, the role of immune cells in NEC remains unknown, and this is a direction for our future research. Ultimately, whether *B. fragilis* inhibits FXR through other mechanisms remains unknown, and we will further investigate this question in our future research.

## Supplementary Material

suppl figure_KGMI_20231361.docx

## Data Availability

Raw mRNA expression profiles and clinical features of the GSE46619, GSE64801, and GSE198372 datasets are available in the GEO database (http://www.ncbi.nlm.nih.gov/geo/). We confirm that all the data in this manuscript are original, and we have access to the raw data files.
