# Supplementary material for: Bacteroides fragilis alleviates necrotizing enterocolitis through restoring bile acid metabolism balance using bile salt hydrolase and inhibiting FXR-NLRP3 signaling pathway
Source: Gut Microbes. 2024 Jul 16;16(1):2379566. doi: 10.1080/19490976.2024.2379566 (PMC11253882; doi:10.1080/19490976.2024.2379566)
Supplement: suppl figure_KGMI_20231361.docx [file KGMI_A_2379566_SM4961.docx]

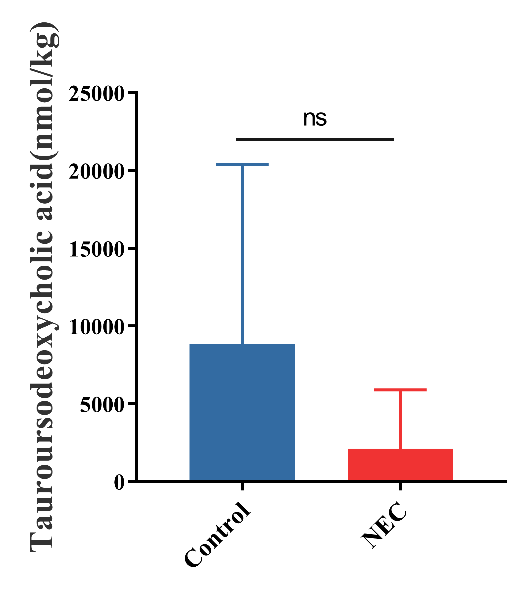


Supplementary Fig. 1 Concentration of TUDCA in the feces of healthy infants and infants with NEC.


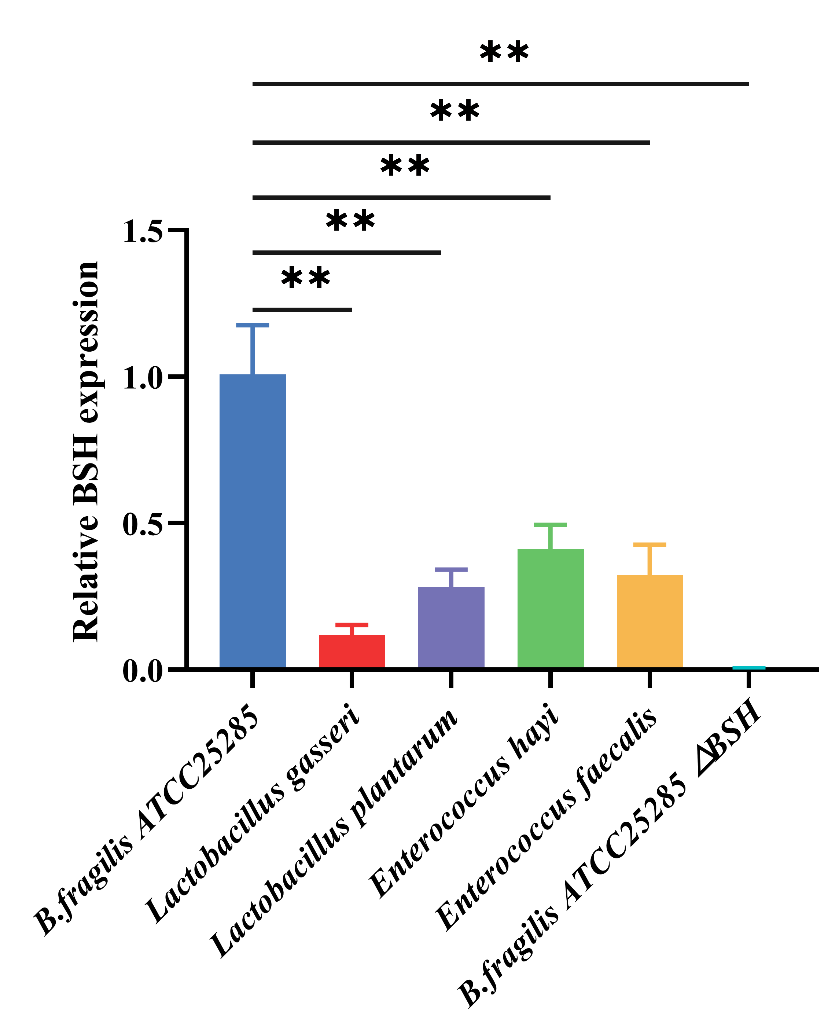


Supplementary Fig. 2 The mRNA level of BSH in 6 strains of bacteria **p*<0.05; ** *p* <0.01.
